# Acid-sensing ion channel 3: An analgesic target

**DOI:** 10.1080/19336950.2020.1852831

**Published:** 2021-01-04

**Authors:** Jasdip Singh Dulai, Ewan St. John Smith, Taufiq Rahman

**Affiliations:** Department of Pharmacology, University of Cambridge, Cambridge, UK

**Keywords:** Acidosis, acid-Sensing Ion Channel 3, analgesia, asic3, inflammation, ion channels, nociception, pain, pH

## Abstract

Acid-sensing ion channel 3 (ASIC3) belongs to the epithelial sodium channel/degenerin (ENaC/DEG) superfamily. There are 7 different ASIC subunits encoded by 5 different genes. Most ASIC subunits form trimeric ion channels that upon activation by extracellular protons mediate a transient inward current inducing cellular excitability. ASIC subunits exhibit differential tissue expression and biophysical properties, and the ability of subunits to form homo- and heteromeric trimers further increases the complexity of currents measured and their pharmacological properties. ASIC3 is of particular interest, not only because it exhibits high expression in sensory neurones, but also because upon activation it does not fully inactivate: a transient current is followed by a sustained current that persists during a period of extracellular acidity, i.e. ASIC3 can encode prolonged acidosis as a nociceptive signal. Furthermore, certain mediators sensitize ASIC3 enabling smaller proton concentrations to activate it and other mediators can directly activate the channel at neutral pH. Moreover, there is a plethora of evidence using transgenic mouse models and pharmacology, which supports ASIC3 as being a potential target for development of analgesics. This review will focus on current understanding of ASIC3 function to provide an overview of how ASIC3 contributes to physiology and pathophysiology, examining the mechanisms by which it can be modulated, and highlighting gaps in current understanding and future research directions.

## The epithelial sodium ion channel/degenerin superfamily

Approximately 1.5% of the human genome encodes ion channels (>400 in total) [1], proteins that are integral to cellular excitability in tissues throughout the body. The importance of ion channels is demonstrated by the fact that they account for 19% and 18% of human protein and small drug targets in the ChEMBL database, respectively [2]. This review will focus on an ion channel family that detects changes in extracellular pH: acid-sensing ion channels (ASICs), and more specifically ASIC3.

**Figure 1.**
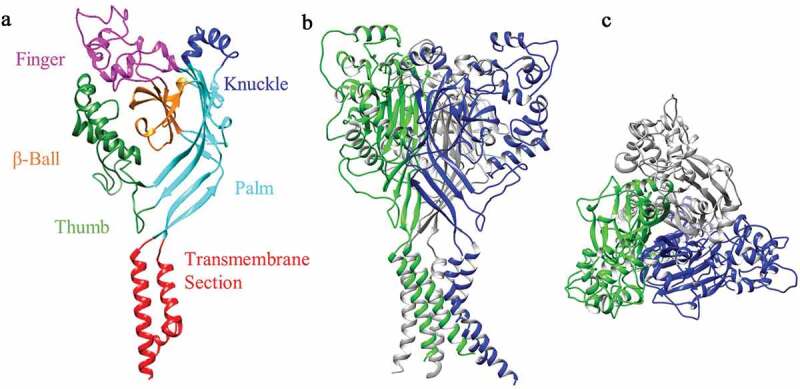
3D Structural Model of rat ASIC3. (a) front view of rASIC3 subunit domains. (b) Side view of homotrimeric rASIC3. (c) Top view from extracellular side. Individual subunits are shown in different colors for A and B. The homology model of rASIC3 was built using the published crystal structure of chicken ASIC1a as template (PDB: 2QTS, PMID: 17,882,215); for details of rASIC3 modeling see Rahman and Smith 2014

The epithelial sodium channel (ENaC)/Degenerin (DEG) ion channel superfamily are selective for Na^+^, showing preference over K^+^ of 100-fold for ENaC and 10-fold for ASIC subunits [3–5]. An additional characteristic of the ENaC/DEG superfamily is sensitivity to the reversible channel blocker, amiloride [6]. In mammals, there are nine ENaC/DEG genes, four encode ENaC subunits (α/β/γ/δ-ENaC) and five encode ASIC subunits (ASIC1-5). Although the nomenclature has been standardized for the ASIC family (Table 1), ASIC5 is still often referred to as Bile Acid-Sensitive Ion Channel (BASIC), due to it not being modulated by extracellular acid [7] and displaying a low amino acid homogeneity of ~30% with other ASIC subunits (by contrast, ASIC1-4 share at least 50% amino homogeneity, Table 2) [7–11]. Due to alternative splicing, the four main ASIC genes produce six ASIC subunits: ASIC1a, ASIC1b, ASIC2a, ASIC2b, ASIC3 and ASIC4 and studies using x-ray crystallography [12] and atomic force microscopy [13] have demonstrated that ASICs form trimers, subunits combining to form homomeric or heteromeric ion channels. To date, a total of 18 ASIC structures are available in the Protein Data Bank (PDB), but all of these are of *Gallus gallus* (chicken) ASIC1a (cASIC1a). Therefore, it should be remembered that all structural information discussed on ASIC3 (Figure 1), both here and in the wider literature, is largely based on the structure of cASIC1a. Like BASIC, not all ASIC subunits form proton-sensitive homotrimers, ASIC2b and ASIC4 both being proton-insensitive [14,15], but able to modulate the properties of other ASICs in heteromeric configurations [16,17]. Of all ASIC subunit configurations, those containing ASIC3 are the most sensitive to protons [18], as well as producing a characteristic biphasic response: an initial transient inward current followed by a smaller magnitude-sustained phase, features that make ASIC3 of particular interest in the context of pain [5]. Human ASIC3 (hASIC3) was first located on chromosome 7q35 (6.4 cRad telomeric) in the late 1990s [19] and there have been limited subsequent studies of hASIC3, and thus, this review will encompass the broader mammalian publications from rat (rASIC3) and mouse (mASIC3) orthologues which share an 84% amino acid identity to hASIC3.

**Table 1.** ASIC Family Nomenclature. Identification of further ASIC subunits has led to the evolution of their nomenclature, finally resulting in the transition to the use of alpha numerical names. NaC – Sodium Chanel, ACCN – Amiloride-Sensitive Cation Channel, BNaC – Brain NaC, BASIC – Bile ASIC, (BL)INAC – (Brain, Liver and) Intestine NaC, DRASIC – Dorsal Root ASIC, MDEG – Mammalian and Degenerin Channel, SPASIC – Spinal Cord ASIC, and TNaC – Testis NaC

Subunit Name	Amiloride Gene Code	Sodium Expression Code	Other/Previous names
ASIC1a	ACCN2	BNaC2α	ASICα
ASIC1b	ACCN2	BNaC2β	ASICβ
ASIC2a	ACCN1	BNaC1α	MDEG1
ASIC2b	ACCN1	BNaC1β	MDEG2
ASIC3	ACCN3	TNaC1	DRASIC
ASIC4	ACCN4	BNaC4	SPASIC
ASIC5	ACCN5	(BL)INaC	BASIC

**Table 2.** Human ASIC Family Homogeneity. Calculated in Clustal Omega using UniProt amino acid sequences

	ASIC1a	ASIC1b	ASIC2a	ASIC2b	ASIC3	ASIC4	ASIC5
ASIC1a	-	79.15	67.46	59.61	50.59	50.41	28.45
ASIC1b	79.15	-	62.17	56.80	49.60	49.90	27.05
ASIC2a	67.46	62.17	-	77.54	50.82	47.33	29.50
ASIC2b	59.61	56.80	77.54	-	47.78	45.40	26.67
ASIC3	50.59	49.60	50.82	47.78	-	46.97	28.90
ASIC4	50.41	49.90	47.33	45.40	46.97	-	25.98
ASIC5	28.45	27.05	29.50	26.67	28.90	25.98	-

## Asic structure and function

**Figure 2.**
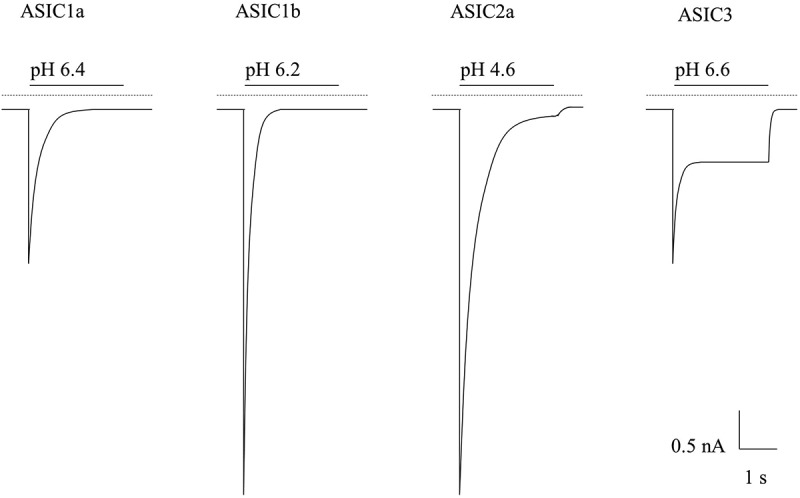
Illustrated Example Current Traces of the pH-Sensitive ASICs. Currents are represented with their half pH maximal activation of a typical rASIC patch-clamp profile. The illustrated example traces are based off published recordings

Early work by Krishtal et al., in 1980, demonstrated for the first time that application of extracellular acid could evoke inward currents in sensory neurones [20], and further work has shown that localized tissue acidification (pH ≤6.0) causes pain in humans [21–23]. Thus, there is a clear link between acidosis, acid-evoked sensory neurone activation and pain. There are a variety of ion channels and receptors that can be modulated by acid, which have been reviewed in detail elsewhere [24], but here we will concentrate on ASICs, which underlie the transient inward currents first recorded 40 years ago. With the exception of ASIC4 and ASIC5, all ASIC subunits show robust expression in sensory neurones [25,26] and their activation by extracellular acid causes transient proton-gated currents that are mainly carried by Na^+^, although ASIC1a homomers also show Ca^2+^ permeability [27]. The ASIC-mediated cation influx leads to neuronal depolarization, which, if of sufficient magnitude to activate voltage-gated sodium channels, can induce action potential firing in nociceptors (sensory neurones tuned to detect noxious stimuli [28]) and ultimately evoke the sensation of pain. Whilst ASICs are gated by extracellular protons, their proton sensitivity can be modulated by other extracellular ions such as Ca^2+^ and Zn^2+^ (discussed in later sections). As illustrated in Figure 2, homomeric ASIC3 and some ASIC3 containing heteromers (especially ASIC2a/3) display a pronounced sustained current in the continued presence of acidity following partial inactivation of the initial transient inward current. This sustained current implies that in the presence of prolonged tissue acidosis, ASIC3 would remain activated, supporting a sustained inward Na^+^ current in sensory neurones causing their membrane depolarization and ultimately prolonged action potential firing. Thus, ASIC3 can effectively transduce the prolonged tissue acidosis occurring in pathological conditions into pain.

Following initial work demonstrating that ASICs are activated by extracellular protons, much research has been dedicated to identifying the region and amino acids that detect protons [27]. In the very first crystal structure of cASIC1a, an acidic pocket situated between the finger-thumb-ball region, was proposed to be critical for proton sensing [12]. However, mutagenesis of residues within this acidic pocket did not fully abolish proton sensitivity for ASIC1a [29,30]. Additionally, rASIC2a is proton-sensitive, whereas its splice variant rASIC2b is not, yet both subunits contain the same 5/6 carboxylate residues of the acidic pocket; site-directed mutagenesis and chimera construction of rASIC2a/2b have identified that the first 87 amino acids after transmembrane domain 1 are of particular importance [16,31]. Furthermore, mutational analysis of the rASIC1a palm domain has identified it to also be an influential component to proton activation, but of less importance than the finger-thumb-ball acidic pocket region [32,33].

In whole-cell recordings of rASIC3-transfected COS-7 cells, the current measured had a half-maximal pH (pH_0.5_) of activation at pH 6.7, a pH_0.5_ steady-state inactivation of pH 7.1 and amiloride inhibited the current at micromolar levels (half maximal inhibitory concentration, IC_50_ = 63 µM) [34]. Mouse ASIC3 (mASIC3) shows similar proton-sensitivity to rASIC3, but naked mole-rat ASIC3 (nmrASIC3), despite sharing >80% amino acid identity with mASIC3 and rASIC3, respectively, is not activated by extracellular protons. Much like proton-insensitive ASIC2b and ASIC4, nmrASIC3 is able to traffic to the cell membrane and modulate the properties of proton-sensitive ASIC subunits [35]. There is an 84% amino acid identity between hASIC3 and rASIC3, the 16% difference accounting for some differences in channel activity, such as hASIC3 requiring only pH 6.0 for sustained current activation, whereas rASIC3 requires a more acidic pH 4.5 [19]. Interestingly, hASIC3, but not rASIC3, has 3 splice variants resulting in differences in the C-terminal domain and one of these, hASIC3a, is activated (albeit to a lesser extent) by alkalinization [36]. Thus, contrary to the function implied by the name *acid*-sensing ion channel, it appears that activation is according to a change in pH, rather than acid-sensing per se. Residues Arg68 and Arg83 located within the base of the palm region of hASIC3a have been implicated in sensing alkalinization, such that if they are mutated the sustained current in high pH is significantly reduced; these residues are absent in rASIC3 which aligns with the fact that rASIC3 is activated only by pH<7.0 [36].

## Pathological tissue acidosis

Nociceptors express ASICs and other acid sensors, suggesting that the ability of the body to detect and respond to acidosis is physiologically important [24]. As will be discussed further on, ASIC3, like many sensory transduction ion channels, can be sensitized by inflammatory mediators and modulated by other proteins, all of which can alter ASIC3’s biophysical properties, including sensitivity toward extracellular protons. Here we will first discuss how tissue acidosis itself can be generated, thus revealing the circumstances in which ASIC3 activation can occur and why it could be considered an appealing therapeutic target in analgesia.

**Figure 3.**
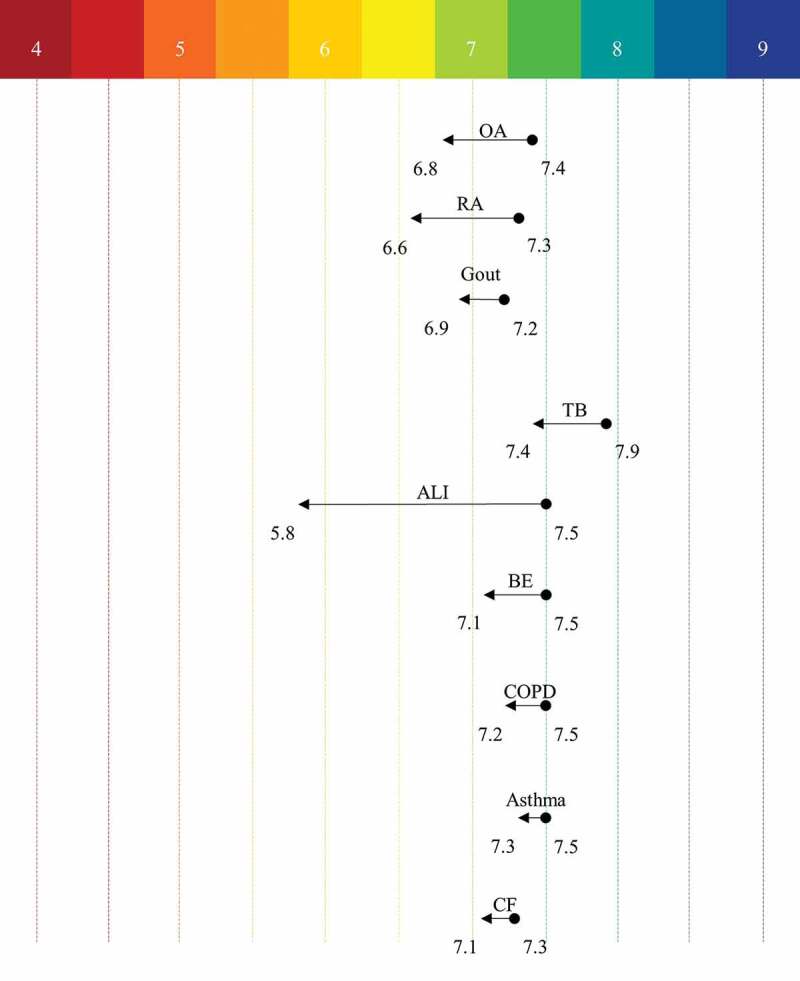
Tissue Acidosis in Human Pathophysiology Conditions. Acidic changes from the normal physiological state to the disease states. Arthritic conditions are local readings (i.e. synovial fluid), and the respiratory conditions are obtained from breath condensate. OA – Osteoarthritis, RA – Rheumatoid Arthritis, TB – Tuberculosis, ALI – Acute Lung Injury, BE – Bronchiectasis, COPD – Chronic Obstructive Pulmonary Disease, and CF – Cystic Fibrosis

In normal physiological conditions, blood plasma pH is pH 7.35–7.45 and is well buffered, but during pathophysiological conditions such as metabolic acidosis there can be marked systemic acidosis due to buffering systems being overcome; a further factor affecting blood plasma pH is temperature, such that in hyperthermia (≥40°C) there can be a decrease by ~0.05 pH [37]. Besides metabolic disorders producing systemic pH changes, there are other conditions that result in local pH changes (Figure 3). For example, inflammation occurs in response to pathogens, trauma, or autoimmune diseases. As immune cells infiltrate the site of injury there is often a concomitant drop in pH, in arthritic conditions, for example, although reported pH values differ, human synovial fluid readings place this acidification for rheumatoid (RA) at ∆ (the difference in pH compared to healthy individuals of) 0.7 pH units [38] and osteoarthritis (OA) at ∆ 0.55 pH units [39]. Gout is a further form of inflammatory arthritis and is linked to uric acid levels. As the joint pH becomes more acidic, urate crystals become insoluble leading to deposition into the joints and researchers examining the urine pH levels of those with gout compared to healthy individuals identified a significant difference (∆ 2.6 units) [40], but a localized pH drop of 0.24 units represents a more reflective change at the disease site [41]. However, it should be noted that not all studies of gout, OA and RA record acidosis, i.e. it has also been reported that synovial fluid from patients with gout, OA and RA can be approximately pH 7.4 [42].

The mucosal lining of the bronchi also experiences changes in pH during different pathological conditions. For example, in pulmonary tuberculosis (TB) a drop of pH 0.5 occurs [43], and airway surface liquid pH in from epithelia in cystic fibrosis (CF) patients is reduced by pH 0.2 compared to that of healthy individuals [44]. Respiratory pH can also be measured through taking breath condensate pH readings and compared to control individuals relative acidosis has been measured in asthma (∆ pH 0.11), chronic obstructive pulmonary disease (COPD, ∆ pH 0.27), bronchiectasis (∆ pH 0.30) [45] and acute lung injury (∆ pH 1.61) [46]. There are other conditions in which acidosis has been measured in rodent models of human disease, e.g. in cardiac ischemia pH drops from pH 7.15 to pH 6.84 [47] and in stroke from pH 7.3 to pH 6.3 [48].

Overall, it is clear that tissue acidosis occurs in a variety of painful conditions and, as detailed previously, ASIC3 has the biophysical characteristics that suggest it can encode prolonged acidosis into nociception and pain. One key aspect of whether or not ASIC3 is able to play a role in different painful, acidic conditions is whether or not it is expressed in the appropriate location, which will now be discussed.

## ASIC3 expression and its pathophysiological roles

The expression pattern of a receptor anchors its function and role in both physiology and pathophysiology, here we will review what is currently understood about the expression and function of ASIC3 in mammals; a summary of ASIC3 expression is provided in Table 3.

**Table 3.** ASIC3 Expression in Different Species. Columns show where expression has been identified, see pathophysiology section for details and references. ASIC-like currents are current recordings which displayed an ASIC3-like profile. IHC – Immunohistochemistry, ISH – In Situ Hybridization, PCR – Polymerase Chain Reaction. G – Guinea Pig, H – Human, M – Mouse (^0^ – unspecified, ^1^ – C57/BL6J, ^2^ – F4, ^3^ – CD1, ^4^ – ‘F2 + 129S2/B6ʹ, ^5^–129S/SvPasCr, ^6^ – BALB/c, ^7^ – ICR) and R – Rat (^0^ – unspecified, ^8^ – Wistar, ^9^ – Hooded Lister, ^10^ – Sprague Dawley, ^11^ – Spontaneously Hypertensive, ^12^ – Wistar Kyoto)

	Northern Blot	Western Blot	qPCR	RT-PCR	ISH	IHC	ASIC-like Current
Retinal Ganglion				R^9^		M^1^	
Dorsal Root Ganglion (DRG)	R^8^,M^1,3^	R^10^		R^8,10^,M^3^,H			
Trigeminal Ganglion				M^7^,H		M^7^,R^10^	
Nodose Ganglion (NG)				M^0^		R^0^	
Cochlear Spiral Ganglion				M^1^		M^1^	
Vestibular Ganglion						R^8^	
Petrosal Ganglion		R^0^	R^0^			R^0^	
Mesensephalic Trigeminal Ganglion						M^3,4^	
Anterior Fungiform Papillae				H			
Tooth Pulp Neurons						R^10^	
Osteoclasts		H		H			
Carotid Body		R^11,12^		R^11,12^,M^0^			
Nucleus Pulposus		H,R^8^		H,R^8^			
Femoral Articular Cartilage						M^1^	
Synovium and Synoviocytes				M^1^		M^1^	
Skeletal Muscle						R^8^	H
Vascular Smooth Muscle Cells		R^0^,M^1^		R^0^,M^1^		R^0^,M^1^	
Pleura and Pulmonary DRG							R^8^
White and Brown Adipose Tissue				M^5^			
Peptidergic Renal Afferents							R^10^
Testis	H						
Monocyte				H			
Dendritic Cells		M^1,6^		M^1,6^			
Spinal Cord	R^0^			H			
Brainstem	R^0^					M^3,4^	
Astrocytes		R^10^				R^10^	
Ear				M^3^	M^3^		
Eye				M^3^			
Organ of Corti				M^3^	M^3^		
Jugular Ganglion				G		R^0^	
Lumbosacral and Thoracolumbar DRG		R^10^	M^1^	R^10^,M^1^	M^1^		R^10^
Hypothalamus		R^10^		R^10^			
Amygdala		R^10^		R^10^			
Caudate Putamen		R^10^		R^10^			
Prefrontal Cortex		R^10^		R^10^,H		H	
Hippocampus		R^10^		R^10^			
Cardiac DRG				M^1^			R^10^
Venoatrium Junction NG						M^3^	M^3^
Gastric DRG and NG				R^10^		R^10^	R^10^
Bladder Smooth Muscle Cells and Urothelium		R^8^	R^8^,H			R^8^	H
Cutaneous DRG		R^8^	R^8^			R^8^	
Gastrocnemius Muscle DRG						R^10^	
Cerebellum				H			
Odontoblast Cell Bodies						H	
Taste Epithelial				R^10^			
Glomus Cells						M^0^	
Bronchial (16HBE14o) and Submucosal (Calu-3) Cells		H		H		H	
Colonic (T84) Cells				H			
Pancreatic (CFPAC) Cells				H			
Submucous and Myenteric Plexus		H					
Intestine DRG		H					

### Central nervous system

All ASICs, including ASIC3, are widely expressed within the central and peripheral nervous systems (CNS and PNS, respectively) [25]. In a study involving (mice and naked mole-rats), ASIC3 appears to be most abundantly expressed in the PNS (dorsal root ganglion, DRG) and represents the predominant ASIC isoform (approximately 10-fold higher expression than ASIC1-4) whilst its expression levels in the CNS (spinal cord and various brain regions) are comparable to or lower than other ASIC subunits [49]. This is in contrast with the early observation showing rASIC3 expression being confined to the PNS [27]. However, several studies now support ASIC3 CNS expression, including in the superior cervical ganglia, spinal cord, brain stem [50] and hypothalamus [51], and recent single-cell RNA-sequencing of various mouse brain regions shows that ASIC3 is expressed in numerous brain regions, albeit higher expression being observed in sensory neurones [25]. Both the hypothalamus and hippocampus play a role in emotional behavior [52,53], and ASIC3^−/-^ mice showed reduced aggression and anxiety-like behavior compared to wild-type (WT) mice [54], i.e. CNS expression of ASIC3 is important to brain function.

By contrast, hASIC3 appears to be more evenly distributed throughout the human nervous system (DRG, spinal cord and brain), with ASIC1a being the most dominant form in DRG [36]. Thus, the relative contribution of ASIC3 in humans compared to rodents with respect to CNS expression might be different. One difference of potential clinical significance is the elevated expression of hASIC3 in GABAergic interneurons of the temporal lobe identified in epileptic patients [55]. This is because inhibition of ASIC3 with the sea anemone *Anthopleura elegantissima* toxin 2 (APETx2), in rat models of epilepsy leads to: shortened latency to seizure, increased incidence of generalized tonic-clonic seizures and significantly decreased frequency of action potential firing in interneurons; thus, ASIC3 may play a protective role through dampening action of potential frequency in post-synaptic neurones [55].

Whereas the role of ASIC3 in epilepsy is likely linked to modulation of interneuron excitability and general CNS activity, studies in migraine models have proposed a role for ASIC3 in mediating pain, where cortical spreading depression is thought to generate ischemia and a concomitant drop in extracellular pH. Indeed, application of acid to the dura in rats produces hypersensitivity to facial stimulation, which is ameliorated by blocking ASIC3, moreover, dura-innervating sensory neurones express ASIC3 [56].

### Peripheral nervous system

Most ASIC3 research to date has been conducted in rodents, with channel expression predominantly in the PNS [49,57], unlike hASIC3 which is reportedly more equally distributed between CNS and PNS. Hence, most published studies have focused on peripheral ASIC3 expression and the accompanying function in pathophysiology.

In rat cutaneous sensory neurones, ~60% exhibit ASIC3-like currents (as identified by sensitivity to the ASIC3 inhibitor APETx2) and responded synergistically to mild acidification, hypertonicity, and arachidonic acid; combinatorial stimuli that also increased nociceptor excitability and produced pain [58]. The group went further to show that ASIC3 expression is increased 24 hours after plantar incision, with inhibition of ASIC3 significantly reducing spontaneous, thermal, and postural pain behaviors [59]. The role of ASIC3 in pain in general is further supported by ASIC3^−/-^ mice displaying a reduced latency to the onset of moderate to high-intensity pain stimuli, in the acetic acid-induced writhing test, hot-plate test and in mechanically induced pain [60]. Moreover, injection of acetic acid (pH ~3.5–4.0) or SL-NH2 (a peptide that causes itch) alone to both ASIC3^−/-^ and WT mice produced no difference in response between genotypes, but a combination of acid and SL-NH2 caused a statistically significant increase in scratching in WT mice compared to ASIC3^−/-^, which was ablated by administration of amiloride; subsequent analysis found that SL-NH2 slows the rate of pH 5.0-induced ASIC3 desensitization which may contribute to itch behavior [61]. Thus overall, these data support the role of ASIC3 in cutaneous pain and itch.

However not all studies support such a key role for ASIC3 in pain. For example, in transgenic dominant-negative ASIC3 mice, Mogil and colleagues [62] observed no difference in sensitivity to heat, but observed an increased response to a mechanical (von Frey and tail-clip test) and chemical/inflammatory (formalin and acetic acid writhing test) stimuli, compared to WT mice, the authors noted this may be attributed to a change in ASIC1a and 2a activity [62]. A further paper also found that ASIC3^−/-^ mice were hypersensitive to high-intensity thermal stimuli compared to WT mice [63]. One possibility for these conflicting findings is differential dysregulation of alternative ASIC subunits, or other sensory ion channels in different mouse genotypes.

### Musculoskeletal system

The musculoskeletal system is a common point of injury, either as a result of traumatic damage or through pathophysiological conditions (arthritis), both being accompanied by inflammation and pain [64]. As discussed previously, acid evokes pain in humans, and a proportional relationship has been shown to exist between the level of acidity and pain response measured in humans when an acidic buffer was applied to the forearm muscles [65].

With regard to specific musculoskeletal conditions, RA is an autoimmune disease often marked by intra-articular decreases in pH, aberrant hyaluronan regulation and destruction of bone and cartilage [66]. Under control conditions, it has been shown that mice express ASIC3 in knee joint synoviocytes, but not in primary afferent neurones innervating the knee joint synovium, whereas post-inflammation (induced by carrageenan), ASIC3 coexpression was observed with protein gene product (PGP) 9.5 and calcitonin gene-related peptide (CGRP) in synovium-innervating primary afferent fibers; furthermore, unlike WT mice, ASIC3^−/-^ mice do not develop secondary mechanical analgesia (in response to von-Frey filaments), but like WT mice do develop primary mechanical hyperalgesia (in response to response to tweezers) [67]. It should be noted that a further study using retrograde tracing has found that ~30% of knee-innervating afferents express ASIC3 under control conditions, which rises to ~50% during inflammation [68], thus afferents innervating non-synovium regions (e.g. bone, menisci, ligaments, etc.) of the knee likely express ASIC3 under control conditions with synovium-innervating neurones also expressing ASIC3 during inflammation. Considering the expression of ASIC3 in both synoviocytes and sensory neurones, development of synovial fluid acidity has the potential to modulate multiple cell types in an ASIC3-dependent manner. Indeed, in fibroblast-like synoviocyte (FLS) cultures isolated from WT mice, exposure to pH 5.5 for 1 hour increased hyaluronan release, by 1.5-fold compared to (physiological) pH 7.4, via increased intracellular calcium [Ca^2+^]_i_ [69]. This could be a potential protective mechanism to compensate for the enhanced ASIC3 expression observed in these cells during inflammation. In addition to ASIC3 expressing FLS cells mediating increased [Ca^2+^]_i_ in response to acidic pH, the addition of inflammatory mediators such as interleukin 1β (IL-1β) caused an ASIC3-dependent increase in the levels of phospho-extracellular signal-regulated kinase (p-ERK), IL-6 and matrix metalloproteinase mRNA, and ultimately FLS cell death, a cascade that was significantly muted in ASIC3^−/-^ mice [70]. This observation of IL-1β modulating ASIC3 function is further confirmed in studies in mice showing that induction of ischemia-related myalgia caused an increase in mechanical hypersensitivity and defensive behaviors, which were both attenuated by knock-down of ASIC3 or the IL-1 receptor 1 [71].

Unlike RA, OA is not an autoimmune condition, but rather involves biomechanical changes in the joint and low-grade inflammation with pain being a key symptom [72]. In rats, using the mono-iodoacetate induced OA model, outside the site of injection there was an increase of both ASIC3 expression in knee joint afferents and weight-bearing pain, effects that plateaued after 3 days, and upon co-administration with the ASIC3-specific blocker APETx2 both changes were diminished [73]. Thus, these results support the findings from RA models showing that the function of ASIC3 appears to be more important in secondary than primary hyperalgesia.

There are many models used to simulate inflammatory pain in rodents, three key acute models being: complete Freund’s adjuvant (CFA), which produces a cell-mediated immune response [74]; carrageenan via a nonimmune-mediated response [75]; and zymosan via an innate immune response [76]. Carrageenan and CFA intra-plantar paw injections have been shown to produce similar degrees of inflammation in WT and ASIC3^−/-^ mice, however, the inflammation does not directly correlate to the degree of hyperalgesia. Whereas both inflammation models produced a statistically significant difference for mechanical hyperalgesia between genotypes (after 24 hours, i.e. ASIC3^−/-^ mice showed less hyperalgesia compared to WT), only carrageenan produced a statistically significant thermal hyperalgesia difference (after 24 hours, i.e. ASIC3^−/-^ mice showed less hyperalgesia compared to WT); furthermore, the ASIC3^−/-^ displayed a reduction in the inflammatory features (granuloma and vasculitis) of muscle compared to WT by ~65% [77], which may perpetuate differences in hyperalgesia between genotypes. In support of these findings, carrageenan-induced muscle inflammation in WT mice is associated with an increase in ASIC3 mRNA in lumbar DRG neurones, and in the same model, ASIC3^−/-^ mice develop only primary, but not secondary, muscle hyperalgesia [78]. In a different model of muscle pain, repeated acid injections into the gastrocnemius muscle caused mechanical hyperalgesia in WT mice, but not ASIC3^−/-^ mice, furthermore, ASIC3^−/-^ mice did not develop cutaneous mechanical hyperalgesia after muscle inflammation whilst heat hyperalgesia occurred at similar levels to in WT mice [79]. Interestingly, the importance of ASIC3 in muscle mechanical hyperalgesia was reinforced by results showing that injecting an ASIC3-encoding virus (and administration of carrageenan) into the muscle of ASIC3^−/-^ mice resulted in rescuing of the development of muscle mechanical hyperalgesia like that observed in WT mice, thus demonstrating a key role for ASIC3 in muscle-innervating sensory neurones in this model [80]. Mechanical hyperalgesia is also a prominent consequence of nerve injury. It has been shown that whilst ASIC3^−/-^ mice showed greater sensitivity to mechanical stimulation compared to WT, in post-spinal nerve ligation of ASIC3^−/-^ mice showed comparable development of mechanical and heat hyperalgesia to WT mice; thus, ASIC3 appears to play a limited role in neuropathic pain [81]. The role of ASIC3 in neuropathic pain has been further evaluated using the cisplatin model of chemotherapy-induced peripheral neuropathy, which causes hyperalgesia and allodynia in rodents [82], increased expression of ASIC3 being observed in DRG neurones innervating the gastrocnemius muscle, alongside mechanical hyperalgesia, which was ameliorated by the ASIC inhibitor amiloride [83].

One potential limitation of ASIC3^−/-^ models is the potential for compensatory upregulation of expression of other acid sensors (e.g. TRPV1 or other ASIC subunits). To address this, a study was conducted employing an artificial miRNA construct (miR-ASIC3) to knock down ASIC3 in a carrageenan-induced muscle inflammation in mice. Using this approach, it was shown that miR-ASIC3 treated mice displayed a reduction in neuronal ASIC3 expression and developed a reduced level of mechanical hyperalgesia compared to WT mice [84].

Another condition in which musculoskeletal pain plays a key component is chronic fatigue syndrome, which is exacerbated post-exercise. In an investigation of post-exercise pain in chronic fatigue syndrome (CFS) patients, there was a strong correlation between post-exercise pain and increased ASIC3 mRNA expression, additionally, baseline ASIC3 mRNA expression was higher in the CFS patients compared to control [85]. In humans, females report greater muscle pain (e.g. fibromyalgia and myofascial pain syndrome) than males, which has also been found to occur in mice, such that female mice develop hyperalgesia for longer 24-hours post-muscle fatigue and injury compared to male mice, an effect not due to estradiol as demonstrated by removal of ovaries (1 week prior to behavior testing), but it remains to be tested if this reduced hyperalgesia in males may be due to testosterone [86].

Globally, one of the leading causes of years lived with a disability (126 out of 195 countries) is lower back pain, which has increased in prevalence from 42.5 million in 1990 to 64.9 million in 2017, standardized by age and sex [87]. A theory explaining the pathophysiology in the intervertebral discs that links to the pain experienced is that of increased lactate production (due to anaerobic glycolysis in the muscle) which leads to reduced pH and ultimately neuronal death [88]. Within the inner core of the invertebrate disc is the nucleus pulposus, which sits in a mildly acidic (pH 6.8–7.2) [89], hyperosmotic and avascular environment, and under these conditions ASIC3 (expressed by nucleus pulposus cells) serves to promote cell survival and lower the activity of the pro-apoptotic protein, caspase-3; ASIC3 was shown to be positively regulated by NGF via p75NTR and ERK signaling [90], a pathway implicated in neuronal diseases and when inhibited, suppresses neuropathic pain [91]. During degeneration of the rat nucleus pulposus tissue, ASIC3 expression is decreased and there is an elevated level of transforming growth factor beta (TGF-β), which has been proposed to downregulate ASIC3 through the SMAD3 pathway [92]. This protective function of ASIC3 in intervertebral disc degeneration is further supported by the observation that culturing human nucleus pulposus cells under acidic conditions leads to increased apoptosis, which can be prevented through the use of amiloride [93,94].

Why might it be that ASIC3 appears to be particularly important in mediating muscle pain? One reason could be that there is a higher expression of ASIC3 in muscle than cutaneous afferents, as has been reported in rats, for example, 83% of ASIC3-expressing muscle afferents also expressed the vasodilatory peptide calcitonin gene-related peptide (CGRP) leading to the hypothesis that these afferents may act as muscle metaboreceptors, triggering pain during insufficient oxygen supply to the muscle, such as might occur during exercise [95]. This hypothesis is supported by femoral occlusion induced by arterial ligation of rat hindlimb muscles (i.e. vascular insufficiency) leading to a significant increase in ASIC3 expression in the surrounding muscle-innervating sensory neurones, suggesting that ASIC3 might contribute to the exaggerated sympathetic and pressor responses observed during arterial occlusion due to lactic acid build up [96].

### Cardiovascular system

As described previously, tissue pH can decrease during periods of cardiac ischemia and humans who suffer a heart attack experience severe chest pain, as do those with angina where coronary blood flow decreases and pH is thought to drop. Studies by Sutherland and colleagues [34] demonstrated that sensory neurones innervating the heart were excited by acid and produced currents with similar biophysical properties to those of rASIC3 [34]. Whilst 90% of rat cardiac-innervating DRG neurones had ASIC-like currents, only 50% of cardiac DRG neurones do in mice, but nevertheless, cardiac innervating neurones isolated from ASIC3^−/-^ mice exhibited a more acidic half-maximal pH of activation compared to WT mice [97]. In rat sensory neurones, ASIC-like currents are sensitized by lactate (a by-product of anaerobic metabolism during cardiac ischemia) due to it chelating Ca^2+^ and the same effect is observed with rASIC3 [98]. Lactate shifts the activation curve of ASIC3-like currents in rat cardiac-innervating sensory neurones, such that they display prolonged sustained currents at more basic pH in the presence of lactate [99]. In addition to lactate, it appears that adenosine triphosphate (ATP) is likely involved in sensitizing of ASIC3, activation of P2X5 by ATP resulting in ASIC3 producing larger responses to acid than acid alone [100]. Furthermore, it has been shown that a combination of acid, lactate and ATP is more effective than each individual substance alone at activating muscle-innervating sensory neurones [101]. Thus, it appears that ASIC3 acts as a coincident detector of lactate, ATP and protons to mediate cardiac and skeletal muscle pain.

At the heart of the cardiovascular system is the requirement of sensing PO_2_ and PCO_2_ in order to regulate breathing and heart rate and a key part of this system are the carotid bodies. ASIC3 is the highest expressed ASIC isoform in carotid body glomus cells, and is considered to be a key mediator of the carotid body response to extracellular acidosis [102], which can result during hypercapnia. Moreover, spontaneously hypertensive rats display enhanced chemoreceptor sensitivity alongside increased ASIC3 expression in carotid bodies, thus further indicating a key role for ASIC3 in carotid body function [103]. Moreover, there is evidence that ASIC3 is also involved in control of blood pressure, as well as regulating breathing rate. For example, ASIC3^−/-^ mice have a lower systolic and diastolic blood pressure, coupled with a slightly, but not statistically significant, higher heart rate, as a potential compensatory role, compared to WT mice [104]. The link between ASIC3 and blood pressure regulation is further supported by the human genetic polymorphism rs2288646-A found in Taiwanese individuals, which is associated with a higher frequency of higher systolic and diastolic blood pressure, allele frequency being 9.7% amongst those with hypertension [105]; however, the effect of this polymorphism on channel function or expression is yet to be determined.

ASIC3-like currents have also been detected in mouse venoatrial junctions nodose neurons, which are low-pressure baroreceptors that detect alterations in central venous pressure and induce the release of atrial natriuretic peptide; in ASIC3^−/-^ mice when blood volume was increased, urine outflow did not increase, thus, blood volume homeostasis is impaired in the absence of ASIC3 [106].

With regard to vascular functions of ASIC3, one contributing factor to migraine, as well as hypertension, is impaired activity of vascular smooth muscle cells (VSMC) [107]. Interestingly, VSMC isolated from cerebral arteries of mice express ASIC3 albeit to a lesser extent than other ASIC subunits [108] and thus ASIC3 is expressed in cells whose dysfunction is associated with migraine and hypertension. Additionally, VSMCs are also implicated in wound healing, a function that becomes reduced upon silencing of ASIC3 (~30% decrease after 24 hours) as chemotactic migration was inhibited [109], hence providing further supporting evidence for ASIC3 playing a role in the regulation of VSMC function.

Lastly, ASIC3 involvement within the vasculature extends to pressure-induced vasodilation, which can lead to ulceration as blood flow is decreased, but ASIC3^−/-^ mice or those treated with diclofenac/APETx2/amiloride show a reduced change (~25% from baseline) in response to local applied pressure (0 to 0.7 kPa), whilst the WT show a peak increased response by 50% at 0.2 kPa [110]; a protective mechanism of ASIC3 in the presence of pathophysiological vasodilatory effects.

### Gastrointestinal system

Of all bodily tissues, the gastrointestinal (GI) system probably encounters the broadest range of stimuli under healthy conditions, as well as being the site of numerous pathological conditions that disrupt bowel function and can give rise to the sensation of pain. We will now discuss the various regions of the GI tract and the diseases affecting GI function.

Furthermore, in vagal and glossopharyngeal sensory ganglia, rASIC3 was found to be coexpressed with CGRP and TRPV1 in jugular ganglia (78% and 62%, respectively), less frequent coexpression was observed in petrosal ganglia (28% and 22%, respectively), and coexpression was rare in nodose ganglia (ND, 6% and 1%, respectively) [111]. As in rat, ASIC3 is expressed in mouse vagal and DRG neurones, with ASIC3^−/-^ mice having a decreased sensitivity of vagal gastro-esophageal tension receptors, colonic mesenteric and colonic serosal mechanoreceptors compared to their WT counterparts [112]. In mouse thoracolumbar DRG neurones, ASIC3 was the least abundant ASIC isoform expressed, but when looking specifically at colonic sensory neurones it was the highest (by 5-fold) [113]. Guinea pigs in comparison to mice display greater levels of ASIC3 (similar to in rats) in vagal esophageal neurones that are putative cell bodies of tension mechanosensors; this interspecies differences also extends to nodose neurones, which follow the same pattern [114]. Thus, ASIC3 is expressed in those sensory neurones compatible with it playing key roles in visceral afferent function and contributing to visceral pain.

When comparing the acid-sensitivity of rat gastric sensory neurones arising from ND ganglia vs. those from DRG, it was observed that the former displayed biphasic ASIC3-like currents, but had a lower (more acidic) half-maximal pH of activation [115]. Given that ASIC3 is expressed by nociceptors, it may contribute to dyspeptic symptoms which include, abdominal pain. In support of this hypothesis, the hyperresponsiveness observed in mice following induction of increased intragastric acidity was greatly diminished in ASIC3^−/-^ mice, the sensory importance of ASIC3 being demonstrated by reduced brainstem activity (measured with c-Fos, a marker of neuronal excitation), and thus ASIC3 can be considered a detector of noxious gastric acid secretion [116]. As expected with ASIC3 activity, mouse ND gastric and gastroesophageal ganglia neurones displayed greater currents with increased acidity, pH 4.0 vs pH 7.0 (control); however, whereas ASIC3 appears to play a role in acid sensation, perhaps important during dyspepsia, TRPV1 appeared to play a greater role in broader chemo- and mechanosensation [117].

A possible role for ASIC3 in Crohn’s disease patients is demonstrated by biopsy samples showing a 2.5-fold increased expression of ASIC3, which was hypothesized to contribute toward visceral hypersensitivity in chronic inflammatory pain [118]. More recently, single-cell RNA-sequencing of colonic sensory afferent neurones has identified ASIC3 to be expressed in 3/7 colonic sensory neurone subsets, thus suggesting that it has a distinct role to play in colonic sensory function [26]. Using transgenic mice to investigate a possible role for ASIC3 in gastrointestinal function and the chronic abdominal pain experienced by patients, ASIC3^−/-^ mice were observed to have a reduction in distention sensitivity of 50%, which would correlate with a reduction in pain [119]. Moreover, zymosan-induced colonic hypersensitivity is blunted in ASIC3^−/-^ mice [120].

### Respiratory system

In rats, 58% of DRG neurones projecting to pleural and pulmonary tissues displayed ASIC3-like currents [121] and rASIC3-like currents have also been reported in vagal pulmonary sensory neurones [122], thus, sensory neurones are well equipped to respond to tissue acidification associated with respiratory disease via ASIC3. In cystic fibrosis (CF), the cystic fibrosis transmembrane conductor regulator (CFTR) is faulty which leads to airway dysfunction leading to viscous mucous and reoccurring lung infections, and a contributory factor in this dysregulation includes a drop in pH [44]. Human lung tissues show co-expression of CFTR and ASIC3, with ASIC3 levels upregulated in CF lung sections associated with the increased inflammation compared to non-CF tissue; additionally, hASIC3 was found to be co-expressed with CFTR in the following human epithelial cell lines, airway submucosal (Calu-3), bronchial (16HBE14o), colonic (T84) and pancreatic (CFPAC) cells, all from parts of the body that have been shown to develop acidic changes in CF patients [123].

The most common chronic respiratory disease globally is chronic obstructive pulmonary disorder (COPD) (3.95% prevalence), followed by asthma (3.6% prevalence) [124], and whilst initial research centered around the involvement of muscarinic, tachykinin and bradykinin receptors, emerging data has implicated ASICs in disease pathology, with a drop in pH being associated with inflammatory airway diseases [125]. A characteristic of asthma is reversible bronchoconstriction leading to a difficulty in breathing and coughing, symptoms which can be induced using citric acid in guinea pigs [126]. In the advanced stages of COPD, there is high prevalence (>75%) of pain, breathlessness and fatigue [127], which can be attributed to the characteristic hypoxia associated with the condition. In rats, three days after chronic hypoxia, the petrosal ganglion has been shown to express elevated levels of ASIC3 (no change in superior cervical or sensory nodose ganglion expression); additionally, chronic hypoxia induces an inflammatory cytokine response (IL-1β, IL-6, and TNF-α) which can also increase ASIC3 expression [128]. Interestingly, the cytokine-induced increase in ASIC3 was prevented by concomitant ibuprofen administration and although ibuprofen inhibits ASIC1a, it has no effect upon ASIC3 [129], thus the effect of ibuprofen is most likely due to its disruption of cyclooxygenase mediated signaling pathways.

### Genitourinary system

One of the alternative names for ASIC3 is the testis sodium channel (TNaC), which came about due to its relatively high expression in the human testis and due to only showing 82% amino acid identity with rASIC3 was initially thought to be a distinct ion channel, however, it has since become clear that TNaC and hASIC3 are the same [130]. Interestingly, a case study documents a middle-aged man with testicular pain coupled with testicular ischemia [131]; and thus it is tempting to propose, considering its high testicular expression, that ASIC3 may have been involved in mediating this pain.

With regard to the excretory side of the genitourinary system, renal-innervating rat DRG neurones displayed ASIC3-like currents (transient and sustained currents at pH 5 and 6), which the authors propose play a role in the vasodilatory effects of inflammatory kidney disease such as hypertension [132]. Further down the excretory route, rat bladder-innervating DRG neurones also display ASIC3-like currents [133] and it is thought that these ASIC3-expressing neurones likely play a role in bladder function because bladder afferents in ASIC3^−/-^ mice no longer respond to extracellular acidification [134]. Moreover, a common form of bladder irritation is cyclophosphamide-induced cystitis and in rats this is accompanied by increased ASIC3 expression in the urothelium and suburothelia plexus, but not in the detrusor smooth muscle, thus further suggesting a role for ASIC3 in bladder function [135]. By contrast, in humans, bladder biopsies show ASIC3 to be expressed in both detrusor smooth muscle and urothelium, increased levels occurring in those presenting with bladder pain [136]. Lastly, painful urination (dysuria) is a common symptom of urinary tract infections (UTIs), where bacteria, such as *Escherichia coli, Enterococcus faecalis* and *Klebsiella pneumoniae*, increase urine pH to make it less acidic. Thus, depending upon the causative bacterium, urinary pH has been reported to rise from a more acidic range of pH 5–7.5 [137], toward the basic range of ≥6.72 in children [138] and ≥6.28 in adults [139]. Considering that hASIC3a is activated by both acidification and alkalinization, and the expression of ASIC3 in bladder afferents, it could well be that hASIC3a plays a role in UTI pain, but this remains to be tested.

### Ears, eyes and mouth

In addition to playing a key role in the somatosensory system, ASIC3 is also expressed in certain sensory organs. For example, in mice ASIC3 is expressed in cochlear spiral ganglion neurones and the organ of Corti [140,141], as well as in the vestibular afferent neurones of rat [142]. With regard to the function of ASIC3 in hearing, ASIC3^−/-^ mice have normal hearing at birth, but go on to develop hearing loss (4 months post birth); however, the mechanism for this early-onset hearing loss and any potential role of ASIC3 is not fully understood [141]. Functioning auditory capability of mothers is essential for them to respond to vocalization of their pups. The auditory frequency of mice is reduced from 2 to 90 kHz measured in WT mice, to 4–32 kHz in ASIC3^−/-^, which was coupled with a reduction in maternal care and pup social development (e.g. reduction in anogenital sniffing, whisker to whisker and push under interactions), phenotypes that were accompanied by reduced serotonergic transmission in the hippocampus, striatum, midbrain and brainstem [143]. Lastly, ASICs may also contribute to the ototoxic action of aminoglycosides, such as neomycin and streptomycin. This is because aminoglycosides reduce the desensitization rate of the ASIC-like currents in mouse spiral ganglion neurones, i.e. Na^+^ influx is maintained for a longer period, and a known side-effect of these drugs is ototoxicity associated with persistent Na^+^ entry [140].

With regard to a role of ASIC3 in vision, in the mouse retina ASIC3 has been shown to be widely expressed, including the rod inner segment of photoreceptors, horizontal cells, amacrine cells, and retinal ganglion cells [144,145]. This appears to provide a protective longevity role because ASIC3^−/-^ mice display retinal rod dysfunction and degeneration [144]. In addition, ASIC3 is expressed by sensory neurones innervating the eye where it may act to detect pH changes, such as the acidification observed in rabbit glaucoma models, where consistent high intraocular pressure (>70 mmHg) leads to a drop in pH (7.32 to 7.03) [146].

Within the mouth, ASIC function has the potential to be involved in both taste and pain sensation. Within odontoblast soma (cells situated on the outer surface of the dental pulp), hASIC3 is expressed at low levels [147]; nevertheless, it may play a role in the transmission of tooth pain [148]. This is because rASIC3 has been observed in 33% of sensory neurones innervating tooth pulp, thus indicating a potential role in dental pain [149]. Similarly, in mice, ASIC3 is expressed in trigeminal ganglion neurones and the periodontal Ruffini, a primary mechanoreceptor, endings [150]. In humans, a case study of two patients showed that they were responsive to bitter, sweet, and salty, but not sour stimuli, an observation that was coupled with ASIC3 being one of a few receptors undetectable in the lingual fungiform papillae of the sour-ageusia patients (others included polycystic kidney disease channels and ASIC1a/1b/2a/2b) [151]. Considering that acid evokes amiloride-sensitive currents in rat vallate taste receptors [152], it can certainly be hypothesized that ASIC3 (perhaps in tandem with other ASICs) is involved in sour taste and thus lack of expression in individuals with sour-ageusia may explain their phenotype.

### Bone and bone marrow-derived cells

Much of the pathophysiological discussion until now has been in relation to the acidosis induced during an inflammatory response, especially in relation to musculoskeletal conditions. However, bone itself can undergo and respond to changes in pH. For example, mild drops in bone pH (pH 7.3–6.9) affect the osteoclast–osteoblast balance which underpins bone integrity, such that acidity shifts the equilibrium to greater bone degradation and active osteoclasts themselves produce an acidic microenvironment [153]. This adaptation to changes in pH in bone in humans has been linked to ASIC3, with expression established in osteoclasts albeit at relatively low levels in comparison to ASIC1 and ASIC2 [154]. Cancer-associated bone pain (CABP), affects >70% of metastatic bone cancer patients and the role osteoclasts and acidification have been implicated in the pain experienced with ASIC3 implicated being involved in transmission of CABP, such that ASIC3 expression is elevated in rat DRGs with tumor-associated CABP [155].

In mouse, ASIC3 expression has been documented in bone marrow-derived dendritic cells, with acidosis leading to upregulated expression of CD11c, MHC class II, CD80, and CD86 via ASICs; this effect was abolished the most by diclofenac which blocks ASIC3 selectively, compared to ASIC1a selective ibuprofen and the nonselective ASIC inhibitor amiloride (see modulator section for more details of inhibitors) [156]. Lastly, ASIC-like currents and expression of ASIC3 has also been documents in both microglia [157] and astrocytes [158].

### Endocrine system

Unlike work on the nervous and musculoskeletal systems, there has been little detailed investigation of how ASIC3 might contribute toward endocrine function. However, in an analysis of 606 Taiwanese individuals, 33 possessed the ASIC3 rs2288646A polymorphism and this was associated with a statistically significantly lower frequency of insulin resistance and lower fasting serum insulin levels [159]. With age, glucose tolerance declines and insulin resistance increases (contributing toward a high prevalence of type 2 diabetes in the elderly population) and interestingly ASIC3^−/-^ mice, as well as wild-type mice administered ASIC3 inhibitors displayed improved glucose intolerance with enhanced insulin sensitivity [160]. In either case, the exact mechanism by which ASIC3 (and the rs2288646A polymorphism) is involved in blood glucose regulation is unknown. Long-term diabetes is associated with diabetic neuropathy, which affects 40–50% of diabetics in their lifetime [161,162]. Considering the role of ASIC3 in pain and that lactate levels increase in response to elevated glucose levels (i.e. after meals) [163], this may add to the potential for ASIC3 sensitization to contribute to pain in diabetic neuropathy.

## Modulation of ASIC3

**Figure 4.**
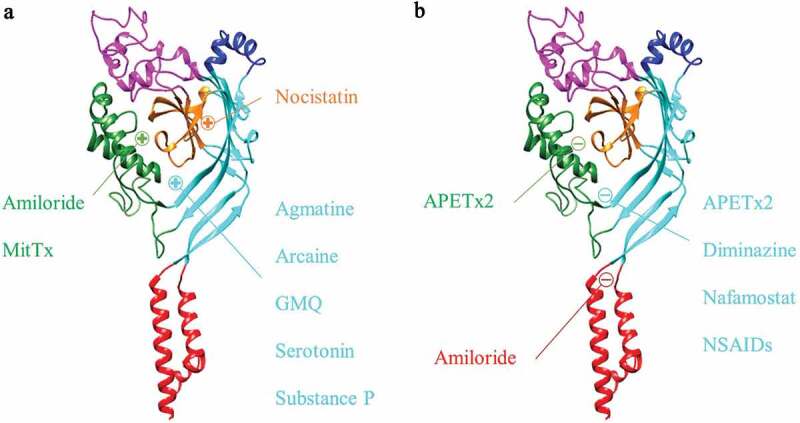
Binding regions of ASIC3 Modulators. Proven (co-crystal structures and mutagenesis) and suspected (in silico) binding of agonists (a) and antagonists (b) to the rASIC3 binding regions. The homology model of rASIC3 was built using the published crystal structure of cASIC1a as template (PDB: 2QTS, PMID: 17,882,215); for details of rASIC3 modeling see [200]

As detailed in the preceding sections, ASIC3 is not only the most highly sensitive ASIC subunit to extracellular protons, but is also implicated in a plethora of physiological and pathophysiological conditions that in many cases are directly or indirectly associated with pain. Consequently, there has been significant interest in developing subunit selective ASIC modulators. To date, 49 endogenous and exogenous (Table 4) ASIC3 modulators have been identified that either cause a change in ASIC3 expression or function; excluding the numerous ligand-based screened synthetic Merck compounds, which are discussed in detail in the Amiloride Analogues section. For some of these modulators, the putative binding sites on ASIC3 have been suggested through in silico modeling and/or mutagenesis studies (Figure 4) and many have been evaluated for their analgesic activity.

**Table 4.** Endogenous and Exogenous Modulators of ASIC3. Categorized into their excitatory/agonist or inhibitory/antagonist effects on the peak transient and sustained currents, data is inclusive of both human and rodent ASIC3. 4-AP – 4-aminopyridine, 5-HT – Serotonin, AA – Arachidonic Acid, ATP – Adenosine Triphosphate, DTT – 1,4-Dithiothreitol, EGCG – (‐)‐Epigallocatechin Gallate, GMQ – 2-Guanidine-4-methylquinazoline, LPC – Lysophosphatidylcholine, NPSF – Neuropeptide SF, SNAP – S-nitroso-N-acetylpenicillamine, SP – Substance P, THP – tetrahydropapaveroline, TPEN – N,N,N’,N’–tetrakis-(2-piridilmetil)-etilendiamina

	Excitatory/Agonist	Inhibitory/Antagonist
Transient	ATP, DTT, Lactate, Reticuline, SNAP, THP, TPEN	α-Dendrotoxin, 4-AP, Amiloride, APETx2, BM-21, Ca^2+^, Cd^2+^, CGA, Cu^2+^, Diminazene, EGCG, Gd^3+^, Hcr 1b-1, Ligustrazine, Mg^2+^, Ni^2+^, NS383, Pb^2+^ [9,223,223], PhcrTx1, Propofol, Thalassiolin B, Zn^2+^
Sustained	5-HT, Chloroquine, FMMRamide, NPFF, NPSF, Sephin1, SL-NH2, SP	Aspirin, Diclofenac Acid, Gastrodin, Salicylic Acid, Tetracaine
Both	AA, GMQ, Guanabenz, Agmatine, Arcaine, Hyperosmolarity, Lindoldhamine, LPC, YPFFamide/Endomorphin-2, YPWFamide/Endomorphin-1	A-317,567, Nafamostat Mesilate, Sevanol, Ugr9-1

### The inflammatory soup

As discussed previously, tissue acidosis commonly develops in inflammation, but protons are just one of a plethora of mediators present in the inflammatory soup that can activate and sensitize sensory neurones; sensitization being mediated by a mixture of changes in nociceptor gene expression and/or post-translational modification of receptors involved in transducing or transmitting noxious stimuli. For example, it has been shown that the following inflammatory mediators lead to increased ASIC3 transcription in DRG neurones: nerve growth factor (NGF, 7-fold alone vs control), serotonin (5-HT, 10-fold), interleukin-1 (IL-1, 5-fold) and bradykinin (BK, 4-fold) with a combined effect of an 8-fold increase [164]. With regard to post-translational modification of ASIC function, protein kinase C (PKC) stimulation by phorbol 12,13-dibutyrate (PDBu) enhanced amplitudes of rASIC3 currents, but only when co-expressed with ASIC2b, this is because only ASIC2a/b, and not ASIC3, can interact with protein interacting with C kinase-1 (PICK-1), which is necessary for PKC modulation [165]. In sensory neurones it was shown that 5-HT and BK, which activate PKC signaling, are able to potentiate ASIC3-like currents [165].

Many inflammatory mediators induce increases in [Ca^2+^]_i_, which activate phospholipase A2 (PLA_2_), an enzyme that cleaves membrane phospholipids to produce arachidonic acid (AA), the precursor to prostaglandin production. Although the roles of prostaglandins in nociceptor sensitization and pain are well understood, AA can itself have effects upon nociceptor function, including upon ASIC subunits, including ASIC3, i.e. 50 µM AA potentiates the pH 6.9 induced activation of the rASIC3 transient current 2-fold [166]. Hyperosmolarity (600 mOsmol kg-1) can also occur in inflammation and combined with AA (10 µM) was observed to potentiate (both transient and sustained) ASIC3 currents in DRG neurones (at pH 7.2) by 148% and 547%, respectively [58]. Lysophosphatidylcholine (LPC), like AA, is also hydrolyzed from membrane phospholipids by PLA_2_ and activates rASIC3 currents at pH 7.4 with a half-maximal effective concentration (EC_50_) of 4.3 µM, an effect that was proposed to involve binding to the non-proton ligand binding site of ASIC3 [167]. There is also evidence for upregulation of nitric oxide (NO) during inflammation and the NO donor S-nitroso-N-acetylpenicillamine (SNAP) has been shown to potentiate the rASIC3 transient phase at 100 µM in pH 6.9 solution, an effect that appears to involve direct nitrosylation, rather than intracellular signaling cascades [168].

ATP is also generated during ischemia and released from cells that lyse during inflammation, and, as mentioned previously, is able to sensitize rASIC3-like currents predominantly via activation of P2X5 [100]. As well as inflammatory cells producing pro-inflammatory mediators, cell death leads to substances entering the extracellular space, including protons, ATP and the extracellular concentration of thiols (such as glutathione and sulfhydryl groups, which can be mimicked using dithiothreitol [DTT]) increase. Like AA and NO, DTT (1 mM at pH 6) also potentiates mASIC3 transient currents by 357%, a phenomenon that persisted upon wash out [169]. The group also observed that the metal chelator N,N,N’,N’-tetrakis(2-pyridylmethyl)ethylenediamine (TPEN) behaved in the similar manner, increasing transient mASIC3 currents by 164% (1 mM at pH 6); it was proposed that DTT and TPEN act on a redox-sensitive site on ASIC3. As reported in studies examining the effect of lactate upon ASIC3, which increases ASIC3 currents by chelating Ca^2+^, reducing extracellular Ca^2+^/Mg^2+^ concentrations directly increase ASIC3-mediated currents [98,99]. Lactate, which is also released in ischemia, (at pH 7.0, 15 mM in vitro) increased the transient amplitudes of rASIC3 by 70%, this amplitude effect was replicated in the absence of lactate with dual reductions of extracellular Ca^2+^ (Δ 0.29 mM) and Mg^2+^ (Δ 0.12 mM) concentrations [98]. Given the lactate amplitude effects were negated by increasing Ca^2+^ (Δ 0.35 mM) and Mg^2+^ (Δ 0.12 mM) concentrations, it is likely that lactate’s mechanism of action is through chelating divalent cations.

### Analgesics

**Figure 5.**
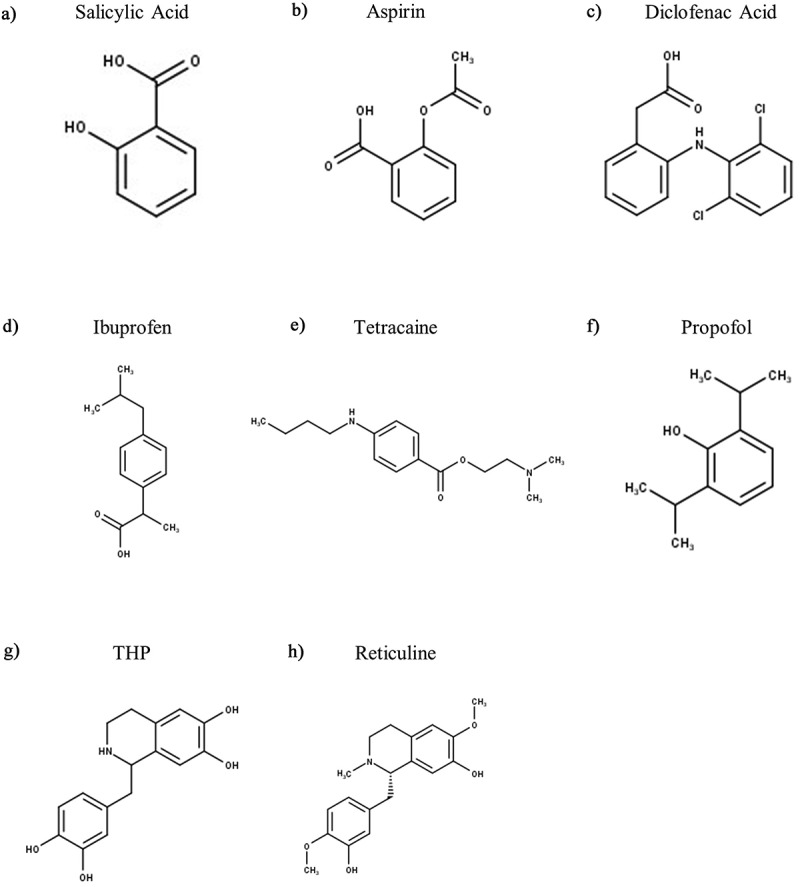
Structures of Analgesics and Anesthetic Modulators of ASIC3. THP – Tetrahydropapaveroline. Structures were produced in MarvinSketch

One class of commonly taken over the counter, and also widely prescribed, analgesics are non-steroidal anti-inflammatory drugs (NSAIDs), which inhibit cyclooxygenase (COX) enzymes to reduce prostaglandin production and thus relieve pain by preventing the sensitizing effects of prostaglandins upon sensory neurones. However, it has also been shown that COX-independent mechanisms exist that contribute to the analgesic effect of NSAIDs [170]. The Lazdunski lab observed that inflammation of rat hindpaws significantly upregulated the mRNA levels of some ASIC subunits (ASIC1a, ASIC2b, and ASIC3) in rat DRG neurones and that this could largely be suppressed by corticosteroids, as well as by some NSAIDS [129]. They further investigated if there were any direct effects of NSAIDs on ASIC subunits when expressed in CHO cells and observed that certain NSAIDs, at high micromolar concentrations, could inhibit ASIC currents in a somewhat isoform-selective manner. Interestingly, salicylic acid (IC_50_ of 260 ± 21 μM, Figure 5a), aspirin (IC_50_ not specified, Figure 5b), and diclofenac (IC_50_ of 92 ± 19 μM, Figure 5c) inhibited the sustained current component of ASIC3 but not the transient component, whereas the ASIC1a current was inhibited by ibuprofen (IC_50_ not specified) and flurbiprofen (IC_50_ of 349 ± 40 μM) only. A range of other NSAIDs, namely piroxicam, etodolac, nimesulide, naproxen indomethacin and acetaminophen had no effect on either ASIC1a or ASIC3. However, subsequent work from Pless lab established that ibuprofen and some of its analogues could inhibit the transient currents of ASIC3 albeit with very weak potency, i.e. ibuprofen had an IC_50_ of 51.3 mM (Figure 5d) at pH 6.4 [171]. Using site-directed mutagenesis, they identified a region comprising the upper of transmembrane helices and extracellular domain β9-α4 loop of rASIC1a to be the main site of ibuprofen binding. Most of the key residues identified for ibuprofen recognition are largely conserved across rASIC1a, rASIC1b, and rASIC2a, but rASIC3 has different residues in two key positions namely Thr in place of Lys76 (K76T) and has Ala in place of a critical Lys (K422A). It is intriguing to note that despite these amino acid differences, ibuprofen’s potency for inhibiting the ASIC3 peak current was comparable to that for inhibiting ASIC1a (IC_50_ of 29.5 mM), which suggests that other binding regions are likely involved.

The structural determinants for the inhibition of ASIC3’s sustained current by salicylic acid, aspirin, and diclofenac remain unknown. In outside-out patches, extracellular application of aspirin or salicylic acid inhibits ASIC3, thus making a direct interaction with an extracellular region of ASIC3 highly likely. In addition, the reversibility of aspirin’s inhibition, as well as inhibition by salicylic acid, suggest that such ASIC3 inhibition by these specific NSAIDS does not involve acetylation [129]. However, employing salicylic acid or high dose salicylates to block ASIC3 and reduce pain is not a clinical option as high doses and/or chronic exposure causes tinnitus and sepsis-like disease [172].

Like NSAIDs, local anesthetics can also be prescribed to relieve pain and act primarily through inhibition of voltage-gated sodium channels, but in a similar manner to certain NSAIDs, some anesthetics inhibit ASIC3. For example, tetracaine (Figure 5e) inhibits ASIC3 transient currents in a pH-dependent manner with an IC_50_ of 9.96 mM (at pH 4.5), with concentrations ≥ 3 mM also partially inhibiting the sustained current (at pH 4.5 and 6) [173]. The general anesthetic propofol (Figure 5f) also inhibits ASIC-like currents in sensory neurones, as well as the transient currents mediated by both rASIC1a and rASIC3, albeit in all cases that only ~20% inhibition was observed [174].

Unlike NSAIDs, opioids are primarily prescribed to control pain, having a very limited role on inflammation. Whereas NSAIDs inhibit ASIC3, tetrahydropapaveroline (THP, Figure 5g), a precursor of endogenous morphine biosynthesis, activates both hASIC3 (EC_50_ of 24.86 mM) and rASIC3 (EC_50_ of 17.23 mM), with another precursor, reticuline (Figure 5h), having a similar activating effect on hASIC3 (EC_50_ of 0.56 mM), all at pH 5.5 [175]. The effect of THP on the transient component was much larger in hASIC3 vs rASIC3 (5.5-fold at pH 5.5), such interspecies variation may be due to a baseline difference in the half-maximal pH for activation of the transient current, rASIC3 = pH 7.20 and hASIC3 = pH 7.67 [175]. Additionally, the endogenous opioid peptides, endomorphin-1 (YPWFamide) and endmorphin-2 (YPFFamide) have been observed to potentiate (both transient and sustained phases) mASIC3 in L-cells (a ﬁbroblast cell line) [176].

A non-pharmacological method of pain control is the use of cryo/heat-therapy, which is used and marketed for relief of muscle pain. Whilst temperature does not directly stimulate nor inhibit mASIC3 activity, reducing the temperature from 34°C to 6°C slowed the rate of (pH 4-induced) desensitization [177]. A similar effect was observed in rat DRG neurones where increasing the temperature (from 22°C to 50°C) accelerated the rate of desensitization [178]; given that the pH used was pH 6.8 and that a biphasic current observed, this effect is most likely attributable to rASIC3. Additionally, it has been demonstrated that the half-maximal activation pH becomes more acidic (pH 6.73 to 6.66) as temperature was increases (25°C to 35°C) [179].

### Amiloride

**Figure 6.**
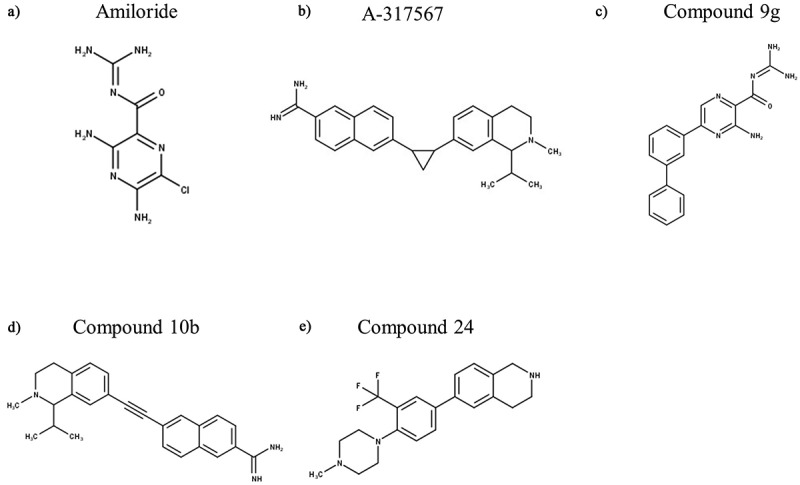
Structures of Amiloride and Other Synthetic Inhibitors of ASIC3. “Compounds” are developed by Merck Research Laboratories. Structures were produced in MarvinSketch

Waldmann and colleagues [5,27] first showed that ASIC3 is sensitive to amiloride (Figure 6a) and its derivatives benzamil and ethylisopropylamiloride (EIPA), all of which are nonselective blockers of ion channels in the ENaC/DEG superfamily; interestingly, these compounds strongly inhibit the transient component of ASIC3 current whilst mildly potentiating the sustained component. Clinically, amiloride is prescribed as an antihypertensive medication with classification as a K^+^ sparing diuretic due to its effects upon ENaC in the nephron [180], thus, amiloride has limited potential as an analgesic due to diuresis being an unwanted side effect. A further disadvantage is that amiloride can cross the blood–brain barrier (BBB) and hence there is also potential for side effects resulting from central inhibition of centrally expressed ASICs. Lastly, the lack of selectivity across ASIC isoforms at comparable concentrations to those that inhibit other ion channels and transporters limits the potential of amiloride directly being used to target ASICs to relieve pain [181–183].

To try and further determine how amiloride interacts with ASICs, Gouaux and colleagues saturated cASIC1a with mitotoxin A (MitTx, PDB 4NTX, see *Toxins and peptides* section) and identified two different locations for amiloride binding: the acidic pocket of the extracellular domain and the fenestration between adjacent subunits, partially occluding the pore region [184]. These sites are conserved between ASIC1a and ASIC3 and are likely to represent the binding sites for amiloride on ASIC3 too. Interestingly, attenuation of ASIC3 currents was observed with a high amiloride concentration (200 μM) at highly acidic pH (pH 4) [5], but the same concentration paradoxically enhanced currents at neutral pH (pH 7) [99]. The structural basis of such enhancement of ASIC3 current by amiloride remains unclear but Xu and Colleges have suggested, using site-directed mutagenesis, that the observed effect was dependent on the integrity of the non-proton ligand-sensing domain in ASIC3 [185].

### Synthetic non-amiloride inhibitors

Abbott Laboratories reported A-317,567 (Figure 6b) as the first small molecule blocker of native and recombinant ASICs, including ASIC3 [186]. This molecule is peripherally active and ~4 times more potent against ASIC3 than amiloride with an in vitro IC_50_ of 9.5 µM (at pH 4.5) and an in vivo analgesic effect from 9 µmol/kg (in rats). Importantly, A-317,567 showed potent analgesic effects in rat models of inflammatory and post-operative pain without affecting the urine output (at concentrations up to 30 µmol/kg), as well as crossing the BBB (<20-fold concentration vs periphery) [186]. Abbott however did not disclose any detailed evolution including the chemical starting point and structure-activity relationship (SAR). The CNS penetration and thus potential for therapeutic and/or side effects was further evaluated, specifically the anxiolytic effect with mixed effects being observed [187].

Several years later, a group of scientists from Merck modified A-317,567 in the region that links the amidine moiety which is found to be critical for inhibition of ASIC3, and an evaluation of a series of indole amidines modified at the 2-position of the indole ring led to the discovery of a potent ASIC3 blocker, “compound 9g” (IC_50_ = 490 nM, Figure 6c), which at 10–30 mg/kg produced similar analgesic effects to naproxen (20 mg/kg) in CFA-induced mechanical hyperalgesia [188]. The same group continued to explore the SAR around A-317,567 and linked the 2-napththimidamide and tetrahydroisoquinoline moieties and produced several derivatives. The compound with an acetylenic linkage (“compound 10b”, Figure 6d) was also highly potent (IC_50_ = 356 nM) ASIC3 channel blocker, which was also found to reverse mechanical hypersensitivity in the rat iodoacetate model of OA, although sedation was noted [189]. Sedation was also observed in ASIC3^−/-^ mice, suggesting that sedation (and thus possibly also analgesia) are mediated independent of ASIC3 inhibition [189]. To identify novel chemical scaffolds against ASIC3, Merck also carried out a high-throughput electrophysiological screening of their proprietary fragment library. From the initial hits, subsequent optimization led to significant improvement of potency (0.7 mM to 3 μM) with retention of good ligand efficiency and incorporation of reasonable physicochemical properties, off-target profile, and pharmacokinetics in rats [190]. The best lead compound (‘compound 24’, Figure 6e) from this fragment-based approach had an IC_50_ of 3.1 μM when measured in vitro, but to date there has been no evaluation of this or other compounds from this (late 2010) report against pain in in vivo models.

Whilst the work from Abbott and Merck has led to the development of small-molecule inhibitors of ASIC3, they have lacked selectivity. Since then, the availability of cASIC1a crystal structures, which were not available at the time of conducted research, could aide a structure-based approach, as opposed to the ligand-based approach employed so far, to develop a selective inhibitor.

### Non-proton ligand activation

**Figure 7.**
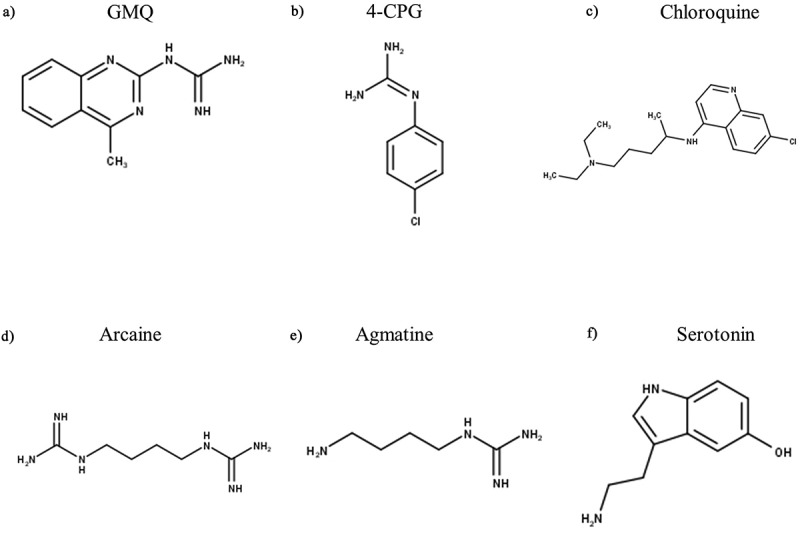
Structures of Non-Proton Ligand Activating Modulators of ASIC3. GMQ – 2-guanidine-4-methylquinazoline, 4-CPG – 4-chlorophenylguanidine. Structures were produced in MarvinSketch

As its name indicates, the primary activator of ASIC3 is protons, but both endogenous and exogenous compounds have been identified that can modulate ASIC3 independently of protons. The first of these to be identified was 2-guanidine-4-methylquinazoline (GMQ, Figure 7a), a small, nitrogen-rich compound, which activates homomeric rASIC3 at 100 µM at neutral pH 7.4 by binding to the non-proton ligand sensing domain encompassing Glu79 and Glu423 [191]. GMQ has an EC_50_ of 680 µM (in rASIC3 at pH 7.4), compounds with related structures guanabenz and sephin1 having an EC_50_ for activation at neutral pH of 67.5 µM and 1220 µM, respectively; in addition, GMQ and guanabenz enhanced transient and sustained phases of acid-activation of ASIC3 [192]. Another guanidine-based compound is 4-chlorophenylguanidine (4-CPG, Figure 7b), which has been shown to increase the ASIC3 half-maximal pH activation from 6.79 to 7.12; but only at a very high concentration of 1 mM [193]. Lastly, a structure similar to GMQ, is chloroquine (CQ, Figure 7c), which has also been shown to increase only the sustained rASIC3 currents at pH 7 (EC_50_ 435.2 µM), an effect largely abolished if Glu79 is mutated and completely abolished if Glu423 is mutated; excitingly, CQ-induced itching behavior in mice was ablated by the ASIC3 blocker APETx2 [194].

Agmatine, (an arginine metabolite, structurally similar to GMQ) and arcaine (an analogue of agmatine) have also been shown to activate ASIC3 at pH 7.4 through interaction with the non-proton sensing domain, also referred to as the palm domain region of the receptor [195]. However, both arcaine (Figure 7d) and agmatine (Figure 7e) had the greatest effect (on both transient and sustain phase currents) at pH 5.0, increasing currents by 20% (EC_50_ = 1.2 mM) and 7% (EC_50_ = 9.7 mM), respectively. Given that inflammation increases both ASIC3 and agmatine expression [196], these in vitro results demonstrate that interaction between agmatine and ASIC3 could have in vivo significance in relation to pain.

Lastly, the non-proton sensing domain has also been recognized as the binding region for 5-HT (Figure 7f), which enhances the sustained current under acidic conditions with an EC_50_ of 41.2 µM at pH 5.0 [197], an effect independent of any signaling through 5-HT receptors.

### Toxins and peptides

**Figure 8.**
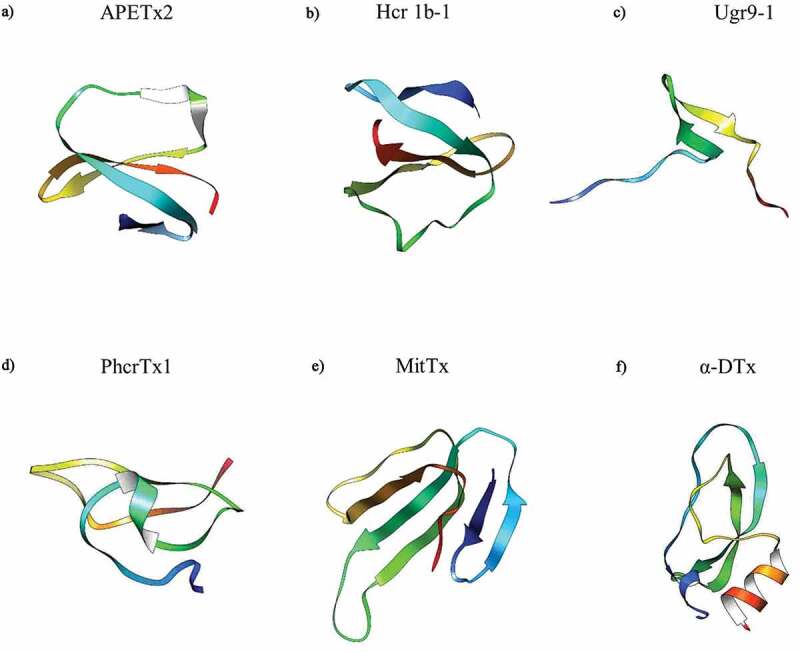
Structures of Toxin Peptide Modulators of ASIC3. α-DTx – alpha-Dendrotoxin. APETx2 obtained from PDB 1WXN, Ugr9-1 obtained from PDB 2LZO, and α-DTx obtained from PDB 1DTX. Hcr 1b-1 (Uniprot: P0DL87), PhcrTx1 (Uniprot: C0HJB1) and MitTx (Uniprot: G9I929) 3D homology models were developed in SWISS-MODEL using the nearest sequence template. Structures were previewed in UCSF Chimera

In addition to endogenous modulators, a range of exogenous poisons and toxins have been identified as modulators of ASIC3. To date, the sea anemone *Anthopleura elegantissima*-derived toxin APETx2 (Figure 8a), is the most potent rASIC3 inhibitor with an IC_50_ of 63 nM; although less potent against hASIC3 with an IC_50_ of 175 nM [198]. Although APETx2 has been used in various animal studies, e.g. subcutaneous administration reduced acid-evoked paw flinching [58] and intraarticular administration ameliorated OA pain [73], being a 42 amino acid peptide makes it inappropriate for systemic use due to it being prone to rapid degradation. Moreover, it is also not selective for ASIC3 homomers, but rather also inhibits certain ASIC3 containing heteromers (1a/1b/2b), as well as inhibiting the voltage-gated sodium channel NaV1.8 [198,199]. Through in silico methods, there have been two predicted sites of interaction, the base of the thumb and the base of the palm [200], but this has not yet been functionally proven.

The sea anemone toxin polypeptide π-AnmTX Hcr 1b-1 (Figure 8b), can be extracted from *Heteractis crispa* and has a similar amino acid identity to APETx2 (49%), also being found to inhibit the transient phase with an IC_50_ of 5.5 µM at pH 4 [201]. Another sea anemone toxin is PhcrTx1 (Figure 8c), extracted from *Phymanthus crucifer*, which partially inhibits acid-evoked currents (≤44%) with an IC_50_ of 100 nM, however, this was conducted in DRG neurones and thus the effect may not be specific to only inhibition of homomeric ASIC3 [202]. Lastly, Ugr9-1 (Figure 8d) is a further sea anemone toxin extracted from *Urticina grebelnyi* and completely inhibited the transient component of hASIC3 currents, at pH 5.5 (IC_50_ of 10 μM), as well as partially inhibiting the amplitude of the sustained component (IC_50_ of 1.44 μM); however, whilst APETx2 inhibits NaV1.8 and PhcrTx1 inhibits voltage-gated K^+^ channels, such inhibition was not observed with Ugr9-1 [203,204].

Conversely, unlike sea anemone toxins, venom from the Texas coral snake (*Micrurus tener*, MitTx, Figure 8e), potentiates ASIC3 (EC_50_ = 830 nM), although this effect was weaker than that observed with other ASIC subunits, ASIC1a and ASIC1b requiring a ∼100-fold lower concentration of MitTx for potentiation [205]; the combined effects of MitTx on multiple ASICs makes interpreting data from neuronal recordings and behavioral studies complicated. cASIC1a crystal structures (PDB Identifiers 4NTW, 4NTX and 4NTY) were made in complex with MitTx (and amiloride), showing that the binding region was similar to that predicted by Rahman and Smith (2014) for APETx2, which is interesting considering the opposing effects of APETx2 and MitTx [184]. Interestingly, α-Dendrotoxin (α-DTx, figure 8f), which comes from the eastern green mamba snake *Dendroaspis angusticeps*, inhibits acid-gated currents in DRG neurones (IC_50_ of 0.8 µM, at pH 6.1) and although the currents were characteristic of ASIC3, this effect should be considered as nonselective ASIC inhibition before further characterization of effects against ASIC3 in a recombinant expression system; there is also evidence that α-DTx inhibits certain voltage-gated K^+^ channels (1.1, 1.2 and 1.6), thus the toxin has nonselective actions [206].

In addition to peptide toxins, certain neuropeptides have also been established as ASIC3 potentiators, but not direct activators. For example, the invertebrate abundant FMRF-amide and mammalian related neuropeptide (NP) FF (NPFF), slowed the inactivation time constant of the pH 4-evoked rASIC3 transient current, but increased the amplitude of the sustained current when applied at 50 µM [207]. A further mammalian peptide, neuropeptide SF (NPSF) displays similar potency to NPFF, again effects only being significant upon the sustained current [208]. A further peptide, RPRFamide (RPRFa), isolated from the venom of the cone snail *Conus textile*, showed similar characteristics to NPFF and NPSF, slowing desensitization of rASIC3 with an EC_50_ of 4.23 µM (pH 6.3), as well as enhancing acid-induced pain [209]. Through site-directed mutagenesis of ASIC3, RPRFa was shown to interact with the lower palm domain (the non-proton sensing domain) of ASIC3 [210] and extensive analysis identified numerous beta sheet regions of the palm domain to be involved in rASIC3 current kinetics: Leu77, Glu79, Gln269, Gln271, Arg376, Ala378, Glu380 and Glu423.

Lastly, the anti-nociceptive peptide nocistatin, which is produced by neuronal cells and neutrophils, has been shown to reduce the speed of activation (49%), amplitude (18%) and the rate of desensitization (32%) of the rASIC3 transient current [211]. Therefore, it is possible that at least some of the anti-nociceptive effect of nocistatin is through inhibition of ASIC function.

### Natural products

There is a growing public interest and endorsement for the use of complementary and alternative medicines for disease management [212]. Commonly prescribed plant-based derivatives include opiates (e.g. morphine from *Papaver somniferum*) for pain relief, digoxin (*Digitalis lanata*) for atrial fibrillation/flutter, and quinine (Cinchona) for malaria/muscle cramps. There are doubtless other pharmacological actions awaiting to be discovered from studying plant-derivatives and the question is, for the purpose of this review at least, do any modulate ASIC3?

**Figure 9.**
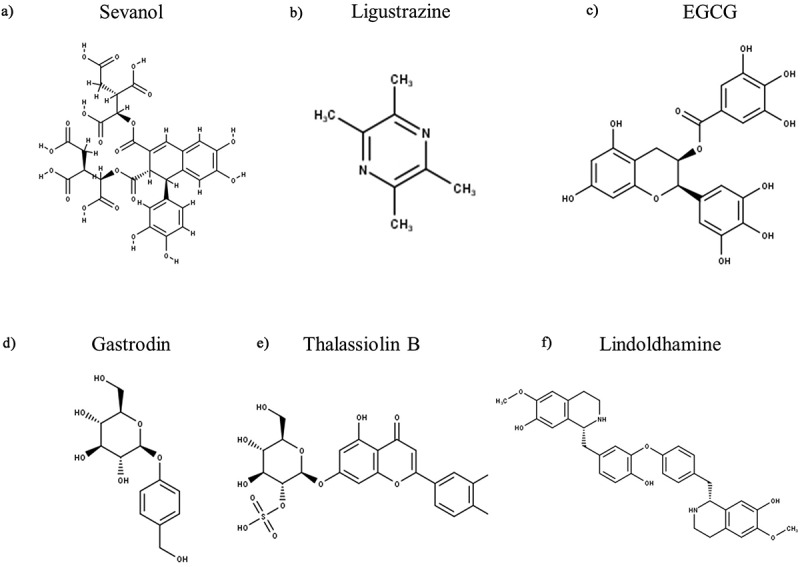
Structures of Natural Product Modulators of ASIC3. EGCG – (-)-Epigallocatechin gallate. Structures were produced in MarvinSketch

The first such compound to be identified is lignan sevanol (Figure 9a), extracted from thyme (*Thymus armeniacus*), which is a reversible blocker of hASIC3, acting independently of the pH stimulus used (from 5.5 to 6.5), and completely blocks the transient component (IC_50_ of 353 µM), as well as partially inhibiting the amplitude of the sustained component (IC_50_ of 234 µM) [213]. In addition to the effects of sevanol on hASIC3, a synthetic analogue, a stereoisomer, and a precursor compound that represents half of the sevanol molecule were tested, all of which showed a marked reduction in inhibition of hASIC3 [214].

Ligustrazine (Figure 9b), is a Chinese traditional medicine used to promote blood flow (menstruation and headaches) and treat inflammatory conditions (boils and carbuncles), which is extracted from the roots of *Ligusticum striatum*. With regard to modulation of ASIC function, ligustrazine decreases ASIC3-like transient currents, at pH 5–6.5 with an IC_50_ of 239.5 mΜ, but had a greater potency against ASIC1a/1b/2a (IC_50_ of 60–130 µM) [215]. By contrast, the natural tea flavonoid (-)-epigallocatechin gallate (EGCG, Figure 9c) is more potent and selectively inhibits ASIC3 with an IC_50_ of 13.2 µM at pH 5; injection of 100 µmol/L also significantly attenuated acid-induced pain responses in mice [216]. Another herbal medicine used for headaches is gastrodin (Figure 9d), a constituent of *Gastrodia elata*, which inhibits the amplitude of ASIC-like currents in DRG neurones (IC_50_ 21 µM, at pH 5.5) and attenuates acetic acid evoked pain [217]. Another Chinese medicine is paeoniflorin (PF), extracted from *Paeoniae alba* and used to treat conditions associated with neuronal damage (i.e. stroke and Parkinson’s disease), which has been shown to reduce the expression of rASIC3 in PC12 cells (a DRG-like cell line) [218].

Furthermore, the extract BM-21, from sea grass *Thalassia testudinum*, and thalassiolin B (a pheloic component of the extract, Figure 9e) are both able to inhibit peak amplitudes of rASIC currents in DRG neurons (at pH 6.1 IC_50_ values were 720 μg/mL and 27 μM, respectively), as well as attenuating mouse pain behavior induced by acetic acid or formalin [219]. In a similar manner, chlorogenic acid (CGA, a natural organic structure common in plants, Figure 9f), inhibits transient acid-evoked currents in DRG neurones with an IC_50_ of 23.5 µM, given rASIC3 is predominant in rat DRG neurone and the nature of the biphasic currents measured, the inhibition observed with CGA is likely due to ASIC3 inhibition; localized injections (1–10 µM) also attenuated acetic-acid-induced pain behavior [220]. In a prior neuropathic behavior study in rats, 200 mg/kg IP injections of CGA produced a similar effect to the analgesic gabapentin (100 mg/kg) [221]; however, the effect of CGA reduced rapidly over time, which may be due to fast metabolism and/or a significant degree of excretion compared to the synthetic compound gabapentin; albeit direct ASIC involvement was not tested.

So far all herbal compounds described are inhibitors, but lindoldhamine (Figure 9g), a bisbenzylisoquinoline alkaloid extracted from *Laurus nobilis*, has been shown to generate sustained currents in both rASIC3 (EC_50_ of 3.2 mM) and hASIC3 (EC_50_ of 3.77 µM) expressing cells at pH 7.8, as well as greatly potentiating acid-evoked transient currents at pH 5.5 and 5.0, for rASIC3 and hASIC3, respectively [222]. Additionally, it was shown that potentiation of hASIC3 transient amplitudes by lindoldhamine (1 mM) was greater under basic conditions (pH 8.5 compared to 7.4).

### Miscellaneous

A variety of cations are present endogenously within the body, as well as used as experimental tools, and thus it is important to understand how ASIC3 is modulated by cations, for example, the inhibitory effects of Ca^2+^ have already been discussed. In addition, the following cations have also been shown to inhibit ASIC3 (and/or ASIC3-like currents): Gd^3+^ with an IC_50_ of ∼2.5 mM [9], Pb^2+^ with an IC_50_ of 8.7 µM [223], high concentration (1 mM) Cd^2+^ and Ni^2+^ [224], Cu^2+^ with an IC_50_ of 14.4 µM [225], and Zn^2+^ with an IC_50_ of 61 µM [226]. Obviously, many of these cations will also modulate the function of other ion channels expressed by sensory neurones and thus interpreting any in vivo activity is complex.

**Figure 10.**
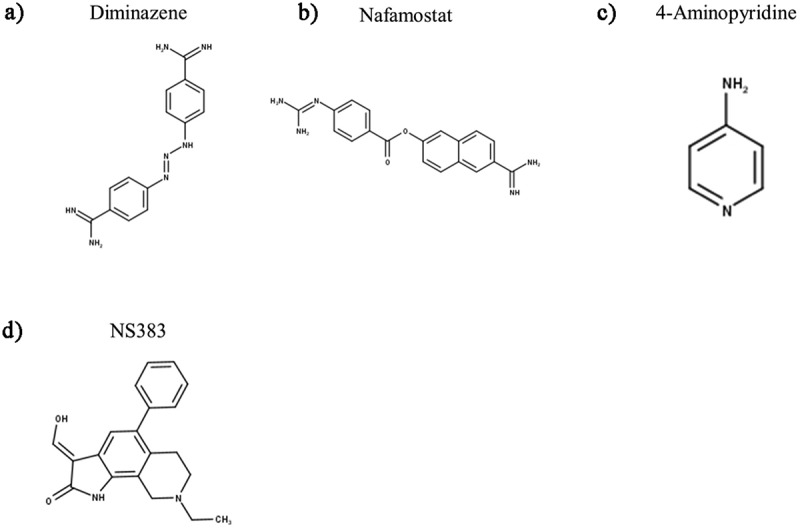
Structures of “Miscellaneous” Modulators of ASIC3. Structures were produced in MarvinSketch

Much like aspirin and diclofenac acid which have their primary action as COX inhibitors, but also directly modulate ASIC3, further potential such re-purposing of medically licensed drugs has been investigated. For example, diminazene (Figure 10A), an anti-protozoan diarylamidine, is a nonselective inhibitor of ASICs with greatest potency against ASIC1b, in silico docking models suggesting that binding occurs at the base of the palm region [227]. In addition, nafamostat mesilate (NM, Figure 10B), is a protease inhibitor licensed for acute pancreatitis also inhibits hASIC3 transient currents (IC_50_ of ∼2.5 µM) and sustained currents (IC_50_ not determined due to current instabilities), but is not selective, also inhibiting ASIC1a and 2a, albeit at 4 and 20 fold higher concentrations, respectively [228]. Moreover, 4-aminopyridine (4-AP, Figure 10C), which can be used to treat multiple sclerosis via blockade of voltage-gated K^+^ channels, also nonselectively inhibits the transient phase of ASIC-mediated currents, with the least effect on rASIC3, reducing amplitude by ~25% (10 mM at pH 5) [229].

Although not used therapeutically, the small compound NS383 [(3E)-8-ethyl-3-(hydroxyimino)-5-phenyl-1 H,2 ;H,3 H,6 H,7 H,8 H,9 H-pyrrolo[3,2-h]isoquinolin-2-one-3-hydroxy-4-mycinide] (Figure 10D) was observed to inhibit acid-evoked rASIC3 transient currents with an IC_50_ of 2.1 µM (pH 5), the inhibition being pH dependent and greater (Δ40%) at pH 6.8; it should be noted that hASIC3 was not inhibited by NS383 and that the inhibitory effect on rASIC1a currents was more pronounced [230]. Interestingly, the in vivo doses of NS383 which produced analgesia (10–60 mg/kg) were not much higher than morphine (3–10 mg/kg, a centrally acting opioid), but much more potent than amiloride (50–200 mg/kg) and acetaminophen (also known as paracetamol, 100–400 mg/kg). Again, NS383, much like NM and Merck’s compound 7d, provides an interesting lead to develop a selective and more potent inhibitor of ASIC3.

In addition to small molecules and various endogenous and exogenous mediators, it has also been demonstrated that other proteins present in the plasma membrane can modulate ASIC3 function, the most well studied being stomatin and stomatin-like proteins. In vitro co-expression of stomatin with mASIC3 leads to reduced transient current amplitude without altering the sustained phase or expression level, compared to cells only expressing mASIC3 [231]. Equally, in vitro transfection of stomatin-like protein 3 (STOML3) with ASIC3 results in reduced current amplitude and sustained currents (at pH 5 and 6) compared to when ASIC3 is expressed alone [232]. The in vivo implications of this have been studied in mice, STOML3^−/-^ mice showing an exacerbated reduction in mechanosensitivity of certain sensory neurones compared to ASIC3^−/-^ mice [233]. Lastly, coexpression of STOML1 with ASIC3 leads to faster inactivation of mASIC3 currents (pH 4 and 6) [234]. Investigation of how stomatin might interact with ASIC3 demonstrated that the C terminus of ASIC3 is important, in particular Leu488+ Leu489, mutation of which causes larger ASIC3-mediated currents and diminished inhibition by stomatin at pH 4.0 [235]. Further analysis has shown that the C terminus of ASIC3 is important for ASIC3-stomatin to physically interact and that transmembrane domain 1 of ASIC3 is critical for the modulation of ASIC3 function by stomatin, as demonstrated by chimera experiments with ASIC1a, which is not inhibited by stomatin [236].

## Areas of interest and gaps in research

One of the drawbacks with the crystal structures that have been employed for in silico virtual screening is that they only represent a snapshot of a fluid channel; moreover, currently only the structure of cASIC1a is available. Due to only the cASIC1a structure being available, any virtual screening protocol against an ASIC other than cASIC1a must employ a homology model and will thus be less accurate compared to when using the actual crystal structure. The amino acid homogeneity between cASIC1a and rASIC3 is 52%, and the two proteins have similar sequence lengths, 527 and 533 residues, and molecular weights of 60.0 and 59.2 kDa, respectively [237]. The amino acid differences may lead to a greater degree of structural differences than predicted by the homology models used in silico, with the regions showing the greatest disparities being 1–85 and 472–542 (the transmembrane regions, which can affect the ability to produce a biphasic current) and 110–161 (finger region, which has yet to be implicated in modulation).

Whilst studies show that in response to an acid stimulus ASIC3 produces a biphasic current and that the sustained element could be linked to prolonged pain sensation, there have not been studies to deduce if this sustained element is directly involved in nociception during chronic pain conditions (constituting >3 months of pain), and unfortunately there do not currently exist any compounds that effectively inhibit only the sustained phased to enable such investigation. There has also to date not yet been an investigation of the contribution of ASIC3 to a chronic pain condition, for example, what is the phenotype of ASIC3^−/-^ mice undergoing destabilization of the medial meniscus model of OA, which develops and is sustained over many weeks. Conducting such a long-term study would enable assessment of whether or not ASIC3 expression changes at different time points and perhaps enable it to be determined as to whether or not the sustained phase plays a role in chronic pain? Also, in humans, is there a role for ASIC3 in chronic pain? At least for acute acid-evoked pain in humans, recent data suggest no role for ASICs [22,238], which contrasts with older literature [21,23], but how this scenario is altered in chronic pain states associated with prolonged tissue acidosis and the presence of multiple inflammatory mediators, many of which sensitize ASIC3, remains unknown.

Finally, hASIC3 has 3 splice variants; thus, greater understanding of how each variant functions and any differential tissue expression are required to determine what, if any, differential roles these variants have. One potential way to investigate this would be to investigate different pain models (both acute and chronic) in transgenic mice expressing the different human splice variants to determine what impact they may have on neuronal excitability and pain.

Fundamentally, what the field really lacks is a selective ASIC3 antagonist that effectively blocks both transient and sustained phases of the current (as well as those derived from ASIC3 heteromers), which could be used in an interventional way to determine the true contribution of ASIC3 to pain and other physiology, and the quest for such a compound remains.
